# The Bilingual Is Not Two Monolinguals of Same Age: Normative Testing Implications for Multilinguals

**DOI:** 10.3390/jintelligence12010003

**Published:** 2023-12-31

**Authors:** Samuel O. Ortiz, Sarah K. Cehelyk

**Affiliations:** Department of Psychology, St. John’s University, Jamaica, NY 11439, USA; sarah.cehelyk22@stjohns.edu

**Keywords:** testing, English learners, test score validity, exposure norms

## Abstract

A fundamental concept in psychological and intelligence testing involves the assumption of comparability in which performance on a test is compared to a normative standard derived from prior testing on individuals who are comparable to the examinee. When evaluating cognitive abilities, the primary variable used for establishing comparability and, in turn, validity is age, given that intellectual abilities develop largely as a function of general physical growth and neuromaturation. When an individual has been raised only in the language of the test, language development is effectively controlled by age. For example, when measuring vocabulary, a 12-year-old will be compared only to other 12-year-olds, all of whom have been learning the language of the test for approximately 12 years—hence, they remain comparable. The same cannot be said when measuring the same or other abilities in a 12-year-old who has been raised only in a different language or raised partly with a different language and partly with the language of the test. In such cases, a 12-year-old may have been learning the language of the test at some point shortly after birth, or they might have just begun learning the language a week ago. Their respective development in the language of the test thus varies considerably, and it can no longer be assumed that they are comparable in this respect to others simply because they are of the same age. Psychologists noted early on that language differences could affect test performance, but it was viewed mostly as an issue regarding basic comprehension. Early efforts were made to address this issue, which typically involved simplification of the instructions or reliance on mostly nonverbal methods of administration and measurement. Other procedures that followed included working around language via test modifications or alterations (e.g., use of an interpreter), testing in the dominant language, or use of tests translated into other languages. None of these approaches, however, have succeeded in establishing validity and fairness in the testing of multilinguals, primarily because they fail to recognize that language difference is not the same as language development, much like cultural difference is not the same as acquisition of acculturative knowledge. Current research demonstrates that the test performance of multilinguals is moderated primarily by the amount of exposure to and development in the language of the test. Moreover, language development, specifically receptive vocabulary, accounts for more variance in test performance than age or any other variable. There is further evidence that when the influence of differential language development is examined and controlled, historical attributions to race-based performance disappear. Advances in fairness in the testing of multilinguals rest on true peer comparisons that control for differences in language development within and among multilinguals. The BESA and the Ortiz PVAT are the only two examples where norms have been created that control for both age and degree of development in the language(s) of the test. Together, they provide a blueprint for future tests and test construction wherein the creation of true peer norms is possible and, when done correctly, exhibits significant influence in equalizing test performance across diverse groups, irrespective of racial/ethnic background or language development. Current research demonstrates convincingly that with deliberate and careful attention to differences that exist, not only between monolinguals and multilinguals of the same age but also among multilinguals themselves, tests can be developed to support claims of validity and fairness for use with individuals who were in fact not raised exclusively in the language or the culture of the test.

## 1. Introduction

Grosjean’s (1989) article, “Neurolinguists Beware! The Bilingual is not Two Monolinguals in One Person”, from which the title of this article is partially adapted, offered a radically different view of individuals for whom early developmental experiences included more than one language. Grosjean’s (1989) analyses highlighted the problem of testing and evaluating bilingual and multilingual^1^ individuals with normative standards drawn from monolinguals in efforts to distinguish language difference from language disorder. Nowhere has this issue been more evident than in the use of standardized, norm-referenced testing, particularly those developed for the purposes of evaluating intelligence and cognitive abilities. Initially, the lack of English proficiency in intelligence testing was flatly dismissed, given the belief at the time that performance was based entirely on genetic makeup (Brigham 1923; Goddard 1913; Terman 1916). And even when some attempt was made to account for language differences (Yerkes 1921), intelligence and English language proficiency remained conflated, especially since vocabulary demonstrated the highest relationship (g-weight) to general intelligence. Since 1974 and the passage of the Individuals with Disability Education Act (IDEA; originally EHCA, PL 94-142), tests of intelligence and cognitive ability have been used as the primary basis for making decisions about whether an individual might have a disability and, as a result, may require special education services (Quintana et al. 2012). The outcomes for monolinguals and multilinguals, however, have been strikingly different. For several decades, multilinguals have been and continue to be disproportionately over-identified as having a disability and placed within the special education setting (Sullivan 2011) as compared to their monolingual peers. Conversely, there has been a concomitant and equally disproportionate under-identification of intelligence in multilinguals and reduced placement in programs for gifted and talented students as compared to their monolingual peers.

Much of the blame regarding the lack of consequential validity in disability identification and other clinical uses of cognitive ability measures has been placed squarely at the doorstep of standardized intelligence tests. Despite an extensive body of literature demonstrating a lack of bias in standardized intelligence tests, the disparities created by their use with monolinguals versus multilinguals remain unabated (Reynolds and Carson 2005), and the reasons for them are elusive. The central thesis of this paper is that the impact that language has on testing has been overlooked primarily because it has been viewed as representing a simple “difference” rather than a highly variable and integral developmental factor directly and powerfully affecting the assumption of comparability. Current research suggests strongly that test performance, particularly between monolinguals and multilinguals, but also among multilinguals themselves, is directly attributable to experiential differences in their respective developmental proficiency in the language of the test (Cormier et al. 2014; Cormier et al. 2022; Ortiz 2019). Tests have routinely been stratified on the basis of a range of variables (e.g., age, race/ethnicity, gender, socioeconomic status, and geographic location), all of which are designed to control for potential differences in performance and create representative samples that ensure comparability. Note, however, that language is not included as a separate variable ostensibly because age itself is a very good measure and estimate of language development within a monolingual population. This premise, however, does not hold in the case of multilinguals for whom developmental experiences exist in two or more languages and in which each language is rarely, if ever, equally well developed. Such differences in language development can be considerable and vary widely even among individuals of the same age. Two children, for example, may both be 10 years old and may share the same heritage language, but one might have begun learning English in kindergarten after 5 years of development in their heritage language only, whereas the other might have begun learning English upon direct entry to the 5th grade after 10 years of development in their heritage language only. Comparing the performance of one to the other is discriminatory, given that they do not share equivalent amounts of development in English or even in their heritage language (Ortiz 2019; Sotelo-Dynega et al. 2013). Likewise, the comparison of either child to a 10-year-old monolingual English speaker is also discriminatory as neither has spent their entire lifetime solely learning English. It is only when these differences are acknowledged, measured, and used for the purposes of establishing comparability in developmental experiences related to language can there be fairness in testing and the evaluation process for multilingual learners (Ortiz 2018; Peña et al. 2018). Standardized testing, in general, whether related to measures of any model of intelligence, language, achievement, neuropsychological domains, or other cognitive abilities, has relied erroneously on the assumption that age serves as an adequate proxy for controlling language development differences. Whereas this holds true for monolinguals, it creates enormous problems with validity and fairness when tests are used with multilinguals. By re-focusing on differences in language development and disentangling age as the solitary control for such differences in normative samples, tests can make great advances in fairness and provide the long-missing validity necessary to truly distinguish the difference from a disorder that may eventually reduce, if not eliminate, current discriminatory outcomes that result from testing multilinguals as if they were two monolinguals of the same age.

## 2. Language Difference as a Practical Concern in Testing

Having returned from Europe, where he had studied under Charles Spearman, Henry H. Goddard (1913) set out to locate “feeble-minded” individuals for the purpose of demonstrating the social utility of his newly translated English version of the French Binet–Simon Scales (Binet and Simon 1905). In so doing, he often found that he needed to rely on an interpreter for administration to the immigrants he evaluated while they stood in line at Ellis Island waiting to be processed through U.S. immigration. And when issues regarding the administration of a test with words and content unfamiliar to or nonexistent in the language and culture of the individual being tested (and who often lacked any formal education) was pointed out to him by his own interpreter, Goddard (1913) summarily rejected it without hesitation:

As Goddard described the scene, a fog hung over New York harbor that day, and no immigrants could land. But one hundred were about ready to leave when Goddard intervened: “We picked out one young man whom we suspected was defective, and, through the interpreter, proceeded to give him the test. The boy tested 8 by the Binet scale. The interpreter said, “I could not have done that when I came to this country”, and seemed to think the test unfair. We convinced him that the boy was defective”.

(p. 105; as quoted in Gould 1996, p. 195)

Goddard’s flat dismissal of language difference as a significant variable that might be affecting the validity and fairness of the testing process is surprising in light of the fact that he was, in that very case, relying on an interpreter to administer his test. His lack of appreciation for the impact that language might be having points to what appears to have been the beginning of a rather simplistic view of the way in which language difference affected performance (only in terms of comprehension of instructions) as well as a tendency to focus on other variables that perhaps carried more social implications (e.g., race). Language, however, and its tendency to significantly disrupt the testing process did not disappear.

In 1917, as part of the contributions to the war effort by psychologists, Robert Yerkes (then president of APA) formed the Committee on Methods of Psychological Examination of Recruits. The Committee developed a multiple-choice, self-administered test based largely on their conceptualization of intelligence at the time and quickly piloted on a group of recruits. The Committee seems to have already possessed notions regarding the impact of cultural and linguistic differences in evaluating intelligence, or else they retrofit these ideas into their report. For example, they couched their mission and purpose as “(1) the detection of low-grade intelligence and (2) the more accurate grading of foreigners” (Yerkes 1921, p. 310). The latter specification certainly suggests that the Committee was aware of the potential inadequacies of intelligence tests with respect to measuring validly and the abilities of those men who were either born outside the U.S., had limited or no education, or who possessed limited, if any, English language development. In fact, after piloting their newly created test, examination alpha, which had been drawn from existing sources, they called upon Edward Thorndike at Columbia University to conduct statistical analyses and provide interpretation of the results. According to Yerkes (1921), in referring to examination alpha, Thorndike “pronounced it incomparably the best battery of group test that had ever been assembled” (p. 320), which must have thrilled the Committee. But Thorndike also explained repeatedly that the validity of his conclusions was defensible only when “assuming that the individual speaks English well and had good opportunity to learn to read English” (Yerkes 1921, p. 319). The issue was so prevalent throughout the results that, ultimately, Thorndike recommended a literacy test to limit the use of examination alpha only for those “men who have had good opportunities to learn to speak and read English” (Yerkes 1921, p. 319). And should someone fail a literacy test, they should then be given an individually administered test of intelligence to ascertain final suitability for military service. 

To what extent the Committee appreciated the issue of language and literacy and how it might affect the overall success of their endeavor is unknown, but they noted that “the proportion of soldiers who were too nearly illiterate to be justly measured by the regular group test was so large that not all could be given an individual examination” (Yerkes 1921, p. 320). The disconnect here between the acknowledgment of language and literacy in English for the purposes of the alpha version of the test, which had been drawn specifically from existing tools for measuring intelligence, and the disregard for language and literacy in determining an individual’s intelligence (the very tools from which the test had been created) is striking. On the one hand, significant attention and concern is placed on ensuring accurate measurement of foreign-born men’s intelligence and fitness for military service while, on the other, there is a complete disregard for the same effect being present in the measurement of intelligence with Goddard’s or Terman’s instruments. This may have been because the Committee viewed their test as primarily a measure for predicting successful military service, which nonetheless relied heavily on intelligence, and not an attempt to assess general, in-born intelligence or describe an individual’s innate worthiness or merit as a human being within society at large. The Committee initially toyed with the idea that perhaps simply giving extra time on examination alpha might provide men of fair intelligence, regardless of English language skills or acculturative knowledge level, the opportunity to score better, but that was not to be. Neither innate intelligence nor the time-limited constraints of the test were shown to moderate performance on examination alpha. Such was the intractable impact of language on test performance that all initial efforts to address the problem of literacy and language differences failed and forced the Committee to look toward “the preparation of a more suitable group test for illiterates” and “ilinguates” (Yerkes 1921, p. 326).

Ultimately, the Committee developed examination beta, a sort of nonverbal type test that, although did not require writing or language ability for responding, was still predicated upon comprehension of verbal instructions accompanied by visual and gestural demonstrations. The Committee found, to their dismay, however, that the test did not equalize the playing field for illiterate and non-English speakers, but they did not make any further attempts to address the issue. To his credit, there is some evidence that Yerkes (1921) may have wanted to do so. In Part III, Chapter 7, toward the end of his 890-page report, he discusses a study examining the relation of intelligence to length of residence in the U.S. This chapter followed the previous discussion wherein he examined intelligence in relation to men born in the U.S. and those born outside the U.S. in Chapter 6, wherein he noted that:

the differences [in intelligence] are considerable (an extreme range of practically two years mental age), and countries fall into two groups: Canada, Great Britain, and the Scandinavian and Teutonic countries all fall in the class interval between 13 and 14 points, whereas the Latin and Slavic countries fall in the class interval between 11 and 12 points.

(Yerkes 1921, p. 699)

**Figure 1:**
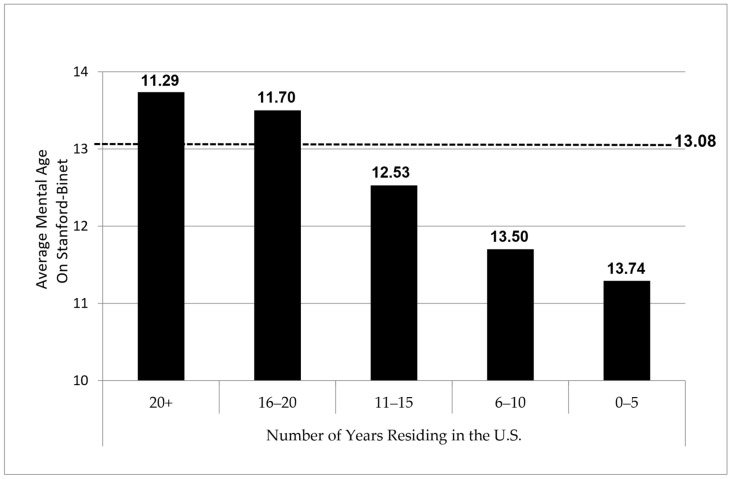
Median Mental Age on Stanford–Binet for Non-English Speaking Army Recruits as Reported in Yerkes (1921).

Why bother to look at the length of residence in the U.S. if the matter of race (using country of birth as a proxy) had already provided the desired and expected eugenics-based answer to differences in intelligence? Perhaps Yerkes’ extensive experience with and meticulously detailed discussions of problems in testing non-English speaking men made him more wary about such facile notions as compared to the typical dyed-in-the-wool eugenicists. Whatever his motivation, Yerkes (1921) investigated the impact of language one step further on the testing of intelligence in non-English speaking men. As in the previous chapter, he again discusses in Chapter 7 the measurement of average mental age^2^ (via individual administration of Terman’s Stanford–Binet) for a population of foreign-born men, all of whom represented the group that had repeatedly performed low due to limited or lack of English proficiency and who had defied improvement on examination beta as hypothesized. But in this analysis, rather than rely on country of birth, Yerkes (1921) instead stratified the sample “by [five] separate units for the first five years of residence and by five-year groups thereafter” (p. 701) on up to 20+ years. Apart from stating that the last grouping represented men who had been in America since childhood, given that the age cap on the draft was 31, Yerkes (1921) offers no explanation or rationale for creating this grouping. It seems possible that Yerkes may well have entertained the heretical notion at the time that perhaps test performance on examination beta was less dependent on race and more a function of differences in exposure to and opportunity for English language development and acculturative knowledge acquisition. Stratification variables are not chosen randomly and are instead selected deliberately and intentionally precisely because they are viewed as likely candidates that may illuminate important causal or explanatory relationships. Moreover, stratification by the length of residence clearly represented an elegant and direct way of evaluating the hypothesis that the intelligence of a recruit might well be due primarily to the amount of time a man had lived in the U.S. during which he could learn English and acquire mainstream acculturative knowledge over time. Not surprisingly, Yerkes’ (1921) results indicated powerfully that as years of residence increased, so did the average mental age of the group. The findings are illustrated by the graph in Figure 1. 

By using the length of residence in this way, whether intentionally or not, Yerkes (1921) was able to show that intelligence could be increased simply by living in the U.S. Not only were the differences between each group statistically significant, Yerkes (1921) also reported an overall increase of 2 years mental age across the groups—a difference he had already described as being “considerable” and “extreme.” He found that men who possessed a minimum of 16 years of residence in the U.S. rose up from the category of feeble-minded and into the normal range (13 years mental age) as established by the Stanford–Binet. Men with even longer lengths of residence in the U.S. pushed median intelligence levels even higher. Yerkes (1921) appears to concede the point and admits, “Apparently then the group that has been longer in resident in this country does somewhat better in intelligence examination” (p. 704). Standing on the very doorstep of the discovery of an incredibly meaningful implication, Yerkes then seems to withdraw from the rather obvious conclusion—that language and acculturative knowledge acquisition are important moderators of intelligence and that these variables are learned, not innate. Instead, he downplays this idea heavily by asserting that “it is not possible to state whether the difference is caused by the better adaptation of the more thoroughly Americanized group to the situation of the examination or whether some other factor is operative” (Yerkes 1921, p. 704). His specification of and reference to the “more thoroughly Americanized group” suggests that he knew language and culture were at the forefront of the short list of variables influencing test performance in this manner. Yet, he was either afraid of the potential ramifications of these findings, which he had previously noted could undermine the validity of testing for any recruit who was not literate and proficient in English, or else he was likely swept up in the wave of eugenics-based arguments of the time and unwilling to swim against the tide. Both may actually be true as Yerkes (1921) did meekly offer the rather ludicrous idea that “the more intelligent immigrants succeed and therefore remain in this country” (p. 704), but his disdain for such a notion is evident by his admonition that “this suggestion is weakened by the fact that so many successful immigrants do return to Europe” (p. 704). That he may have been conflicted but still reticent to engage any further in the debate surrounding nature vs. nurture arguments of intelligence is revealed in his concluding comment, “At best we can but leave for future decision the question as to whether the differences represent a real difference of intelligence or an artifact of the method of examination” (Yerkes 1921, p. 704). Perhaps surprisingly, by 1921, Yerkes and the Committee had already identified the very issue that subsequent test development needed to address if it were to succeed fully in establishing fair and valid measurement of intelligence for limited or non-English speakers. For reasons that can only be speculative, the issue was never taken up seriously, and the entire direction of testing of multilinguals missed an early opportunity to alter test development for the better with respect to multilinguals.

## 3. Language Difference as a Comprehension Issue in Testing

As tests continued their development into the modern era, the issue of language difference and its impact on comparability and validity languished in the background. Even when it was addressed, language was viewed largely as something which prevented comprehension of test instructions, not something that violated the assumption of comparability used in norm sample creation and stratification. The unfortunate early focus on genetically-driven differences in intelligence meant that few psychologists likely felt compelled to expend much time or resources investigating language and knowledge factors in the testing of multilinguals. Those who did (Sánchez 1934) found it difficult to publish their work given that it argued against the prevailing ideology based on genetic explanations and must, therefore, be presumptively wrong. Moreover, it may have been believed that the adoption of the dual structure combining verbal and performance items for testing intelligence and other abilities was already sufficient with respect to accommodating language differences, particularly if one only used performance measures. 

Testing Nonverbally. In his original Wechsler–Bellevue Scale (Wechsler 1939, 1946), the organization adopted by David Wechsler reflected, almost without modification, the same verbal and performance task structure pioneered by the Army Mental Tests during WWI. This is not surprising as Wechsler had served as a young lieutenant under Col. Yerkes on the Committee right alongside Terman, Goddard, Brigham, and others. As such, he was no doubt aware of issues regarding testing with multilingual populations, but like most other psychologists, he was probably happy to let the matter of fairness and validity in testing them simply rest on the use and application of performance-oriented tasks. This naturally gave rise to a clinical tradition for measuring the abilities of multilinguals that constituted administration of only the subtests that comprised the Performance IQ (PIQ) that he had adopted from examinations alpha and beta for his own scales of intelligence. This clinical practice represented a straightforward, but still slightly off point, extension of the work pioneered by Yerkes (1921) and the Committee. Many comprehensive intelligence batteries still mirror this structure today by providing sufficient performance-type tests with which to calculate and derive a more nonverbal measure of intelligence. For example, the KABC-II (Kaufman and Kaufman 2004) offers the Nonverbal Index (NVI), the Stanford–Binet 5 (Roid 2003) provides a nonverbal subtest in each domain measured as well as a broad nonverbal index, and the Differential Abilities Scales II (Eliot 2007) offers a nonverbal/nonverbal reasoning (Gf) composite as well as a broader Special Nonverbal (SN) composite.

As the linguistic diversity of the U.S. population has increased, particularly those of school age, combined with the passage and subsequent revisions of IDEA (2004), there has arisen an entire endeavor devoted to the creation of nonverbal test batteries as a method for conducting evaluations of individuals whose development encompasses more than one language. Examples include the Universal Nonverbal Intelligence Test-Second Edition (UNIT-2; Bracken and McCallum 2016) and the Leiter International Performance Scale—Third Edition (Leiter-3; Roid et al. 2013), among many others. Batteries of this kind intentionally restrict the manner and nature of measurements to tasks that reduce the need for comprehension of instructions delivered orally or responses that require expressive language ability. This limits, of course, the abilities that can be measured, especially those related to actual language functioning. In a sense, nonverbal batteries, by definition, do not account for the contribution of language as it may be related to both general intelligence as well as its relationship to presumptive causes of learning difficulties in school, such as reading and writing problems.

On the whole, nonverbal testing has shown some success in reducing differences in performance and rightly so, since it attempts to work around language issues by reducing its impact on evaluation—much in the same way as examination beta was designed to do, but it does not erase these differences completely, and the measurement of nonverbal abilities has its own set of limitations (Ortiz 2019). For example, communication (whether oral or gestural) and comprehension must necessarily remain a part of the testing equation even when no language response is required on the part of the examinee (Lohman et al. 2008). Moreover, not all abilities can be measured nonverbally, particularly those that are central to the development of language and literacy skills that form the bulk of academic instruction, such as Gc (comprehension knowledge—lexical knowledge, communication ability, general verbal information, listening ability, language development, grammatical sensitivity) and Ga (auditory processing—phonetic coding, speech–sound discrimination, etc.). Nonverbal tests may also limit the evaluation of verbal and quantitative reasoning ability and underrepresent them to the extent that it reduces the validity of obtained test scores and their relation to academically related fluid reasoning (Braden 2000). 

Nevertheless, it is important to recall that Yerkes (1921) had concluded that examination beta was no panacea when it came to evaluating the intelligence of multilinguals and that “the non-English-speaking individual is penalized to some extent in beta, even though not to the same degree as he is in alpha” (p. 383). Although the performance of multilinguals on nonverbal tests did not attenuate performance as much as on verbal tests, the impact on the measurement of intelligence did not automatically disappear. In 2008, Lohman and colleagues examined the validity of three nonverbal tests for the purpose of identifying academically gifted multilingual learners with the Raven Standard Progressive Matrices (Raven et al. 1996), the Naglieri Nonverbal Abilities Test (NNAT; Naglieri 1996), and the Cognitive Abilities Test-Form 6 (CogAT; Lohman and Hagen 2001). The authors noted that “nonverbal reasoning tests can reduce the amount of construct-irrelevant variance in test scores for nonnative speakers by reducing the impact of language” (p. 276), but this did not mean they could reliably and validly discern the intelligence of limited or non-English speakers. Lohman et al. (2008) found that “First, and most important, we observed substantial differences between the nonverbal test scores of ELL and non-ELL children, both on average and in the proportions of these children at different points in the score distributions” (p. 290). They later concluded that “one cannot assume that nonverbal tests level the playing field for children who come from different cultures or who have had different educational opportunities” (p. 293).

Testing in the Heritage Language. Although there are currently more than 350 languages spoken in the U.S. (U.S. Census Bureau 2015), Spanish fully represents 70% of those who are not monolingual English speakers. It is no surprise then that initial efforts to increase comprehension in testing were and continue to be rooted in the development of Spanish language versions of existing tests in English. In 1951, the Psychological Corporation released an experimental version of the Wechsler Intelligence Scale for Children (WISC; Wechsler 1949) called the Escala de Inteligencia Wechsler para Niños (EIWN; Wechsler 1951), which was adapted by Pablo Roca for use with children in Puerto Rico. The EIWN was a direct, unmodified Spanish translation of the WISC and was published primarily for the purposes of research as it did not contain any norms specific to Spanish speakers (López and Weisman 2004). Later, there emerged three Spanish-language versions which followed the revision of the WISC into the WISC-R (Wechsler 1974). These included: the EIWN-R (Wechsler 1983) from the Psychological Corporation, which again was a direct and literal translation of the English version issued without any accompanying norms for Spanish speakers; the WISC-RM (Palacio et al. 1984), which was translated and adapted on a research sample of children in Mexico; and the EIWN-R-PR (Wechsler 1992), which was the official adapted and normed version based on Puerto Rican children. Since then, there have been Spanish versions of the battery for most revisions, including the latest version wherein the WISC-V Spanish (Wechsler 2017) is the most current incarnation. As with some prior versions, the WISC-V Spanish includes norms for use with Spanish speakers; however, the recommendation is that it is only valid for those who have been learning English for less than five years. Those with more English exposure are advised to use the English version of the test. Of course, although the WISC-V Spanish represents a sample of multilingual learners, there is no control for differences in Spanish language development among children of the same age—something that again reflects a very narrow and limited understanding of the issues involved in testing multilinguals.

Due to the prevalence and extent of the Spanish-speaking population in the U.S., other test publishers have followed the Psychological Corporation’s lead in offering Spanish language translations of their own tests. One current example with a long history of Spanish adaptations is the Batería-IV (Woodcock et al. 2019), which is a mostly parallel translation of the Woodcock-Johnson IV: Tests of Cognitive Ability (Schrank et al. 2014) but normed primarily on monolingual Spanish speakers from Spanish-speaking countries around the world. As an alternative to the translation and adaptation of the entire intelligence battery, other authors and publishers have instead offered Spanish translations of their test’s instructions for administration and practice items while maintaining English for the presentation of actual test items that are scored so as to preserve validity in use of the English-speaking norms. Both the KABC-II (Kaufman and Kaufman 2004) and the DAS-II (Eliot 2007) are examples of intelligence batteries that have adopted this methodology. 

Much as with nonverbal test development, translation of tests into other languages, notably Spanish, has met with its own set of difficulties. The basic problem is that it has already been argued that multilinguals are not monolinguals and that they vary considerably in terms of their exposure to and development in English. In the same vein, when tests are developed for use in the U.S., it cannot be presumed that two individuals of the same age have both had the same amount of development or education in their heritage language. More than this, it cannot even be assumed that two people who speak the same language by name (e.g., Spanish) actually speak the language in the same way or that they have been exposed to cultural elements that are the same. 

Test developers have either ignored or misunderstood this issue in various ways. For example, when creating translated versions of existing tests, Spanish is often treated as if it is a monolithic language—that is, that it is the same for all people from all countries and all cultures who use and speak it. This is incorrect as the Spanish used in Mexico differs from that used in Puerto Rico, which itself differs from that used in Spain. Vocabulary words, phrasing, formality, pronunciation, phonemes, and more can vary widely among individuals in Spanish-speaking countries (Ortiz and Oganes 2022). Yet, the use of translation and back-translation methods effectively creates a consensus type of Spanish, which is actually a language spoken nowhere in the world and can feel unnatural to those hearing it. The acquisition of acculturative knowledge affects test performance in a similar fashion as the cultural artifacts, which will invariably appear on a test, are a function of the culture in which the test is created. Not all Spanish-speaking countries share the same cultural elements any more than they share the exact same language, and their acquisition remains dependent on an individual’s exposure to and experience with them (Ortiz and Oganes 2022). This means that in many cases, neither the language of the test nor the acculturative knowledge that it employs is acquired innately merely because it is created in a language like Spanish. Parallels to these issues are evident even in English. For example, African Americans who use African American Vernacular English (AAVE) at home and in the community but are taught and required to use Standard American English (SAE) in school are, in effect, multilingual, a concept embraced by Oakland Unified School District in California in 1996.

**Figure 2:**
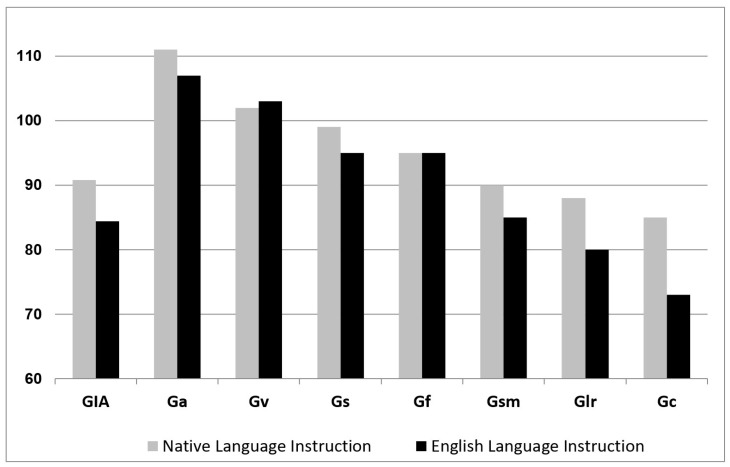
Mean Batería-III GIA and Broad Domain Scores for Bilingual, Hispanic Children in Native Language and English-only Educational Programs as Reported in Esparza-Brown (2007).

An excellent example of the impact that multilingualism has even on tests developed in other languages, such as Spanish, can be seen in cases where the norms are based on monolingual speakers of that language. Consider that when such tests are created for use in the U.S., the use of monolingual Spanish speakers to construct the norms ignores the fact that individuals in the U.S., particularly students, are invariably exposed to English and are required to learn it. They are, therefore, always set forth on a path toward some level of bilingualism and have left their monolingual Spanish speaker status behind. This line of inquiry remains grossly under-researched, but what little is available highlights the similar manner in which acquisition of language and acculturative knowledge affects test performance for multilinguals in the U.S. Figure 2 depicts results from one such study (Esparza-Brown 2007) on bilingual, Spanish-speaking children in the U.S. whose intelligence was tested with the Batería-III (Muñoz-Sandoval et al. 2005) and where a unique and enlightening grouping variable not found in other investigations was utilized—instructional programming in the native language vs. instructional programming in English only.

Not unexpectedly, Esparza-Brown’s (2007) results show a similar pattern as Yerkes (1921) in that performance tests that measure abilities with reduced reliance on age-expected heritage language development (e.g., Gv—Visual Processing, Gs—Processing Speed) is better than performance on tests with reliance on or that are direct measures of age-expected heritage language development (e.g., Glr—Long-term Storage and Retrieval, Gc—Comprehension Knowledge) (Esparza-Brown 2007). These findings are also highly informative in that they demonstrate the proportional impact of language differences on performance, which can be seen in the reduced level of decline in the group receiving native language instruction as compared to the decline for those receiving English-only instruction (Esparza-Brown 2007). Maintenance of the heritage language for instruction assists in maintaining better performance, albeit as compared to the mean of the test that is based on monolinguals, performance remains well below that of monolingual Spanish speakers and highlights the problem once again with monolingual norms applied to multilingual individuals. One other important issue found within these results concerns performance in the area of Ga—Auditory Processing, where testing in English generally produces results that are moderately or more attenuated. In contrast, Esparza-Brown (2007) found that Ga produced the best performance across all measured domains, unlike what is seen in English, which may appear to be surprising but actually highlights another equally problematic issue in the translation and adaptation of tests into other languages—specifically, that some aspects of language cannot be translated directly from one language to another. English is considered, for example, an opaque language because the correspondence between its morphology and phonology is rather low in comparison to other languages. Spanish, on the other hand, is considered a transparent language due to its high correspondence. This means that the application of phonetic coding and phonemic awareness abilities are significantly easier to develop and master in Spanish than in English. Languages are not comparable in every way, and therefore, translations cannot account for basic, structural differences among languages that can and do change the nature and difficulty of a particular task. Simple words in English may have no direct translation in another language. The long “a” phoneme in English does not exist in Spanish, and the trilled “rr” in Spanish does not exist in English. And an individual’s experiences with the development and acquisition of any of these aspects of language will depend entirely upon the amount of time, exposure, and experience they receive in that language—whether alone or in conjunction with the presence of another or even more languages. Norms based on monolingual speakers of any language will thus remain inappropriate for individuals who have been exposed to and are expected to learn another language—the bilingual is not two monolinguals of the same age.

Whereas the preceding approaches in test development appear to represent, at least on the surface, a significant amount of effort over the years in resolving the problem of testing individuals who are not monolingual native English speakers raised in the U.S. cultural mainstream, the reality is that none of them have proven to be wholly satisfactory, particularly when attempting to measure a comprehensive range of abilities that includes language and literacy. Irrespective of attempts to either avoid language altogether or efforts to establish equivalency of a battery in another language, current attempts to measure the intelligence and abilities of multilinguals continue to ignore the manner in which developmental differences in language and acculturative knowledge lead to differences in performance. Perhaps semantic issues have confounded our understanding, but it must be repeated that language difference is not the same as language development and that cultural difference is not the same as acculturative knowledge acquisition. What does affect test performance directly and profoundly is the difference in the amount of time and opportunity for the acquisition and development of English and acculturative knowledge an individual possesses as compared to others of the same age who were raised with English only and within the cultural milieu that gave rise to the test. 

Whether by intention or chance, Yerkes’ stratification of recruits by length of residence in the U.S. clearly highlighted the problem that intelligence clearly increased as a function of opportunity for learning the English language and acquiring specific acculturative knowledge. More exposure to and more experience with English and mainstream culture (by virtue of living in the U.S.) meant better performance on intelligence tests and strongly argued against innate views of ability. Yerkes did not explore the issue any further, but had he done so, it is likely he would have also observed that individuals of the same age can each possess vastly different levels of English language development and literacy. Two individuals of the same age and who have been exposed to the same two languages will not necessarily (or even commonly) demonstrate the same degree of development in both languages compared to each other. One may have more experience and development in the heritage language and less so in English, whereas the other may have the exact opposite. Comparing their performance on any test, whether nonverbally, in the heritage language, or in English, is potentially discriminatory as multilinguals are not automatically comparable in terms of language development simply because they are of the same age, speak the same languages, or were born at the same time in the U.S. Every individual learning English as an additional language or receiving education in a language that is not well supported in the home will be penalized in some way because they are neither monolingual nor necessarily bilinguals with the same level of development. 

## 4. Language Difference as a Developmental Variable in Testing

The fact that both language and acculturative knowledge acquisition are developmental and acquired in a predictable and known age-based sequence means that they do not interact with test performance in ways that create inherent test bias. This is why reliance on predictive validity, for example, as an index of bias, has been so pernicious in undermining efforts to advance fairness and equity in intelligence testing. Any test that measures any ability that may be necessary for success in any endeavor demonstrates predictive validity without any inherent bias precisely because it is measuring the skills, knowledge, or ability necessary for such success. Bias occurs mainly when it is mistakenly believed that the performance on the test in the first place is indicative of innate qualities rather than circumstantial factors, particularly differences in language development and acculturative knowledge acquisition. 

Valdés and Figueroa (1994) attempted to explain this issue by showing that “cultural bias will not be found as Jensen (1974) and Sandoval (1979) expected. Rather, it might be found throughout the test depressing all or most items (the entire sequence of the incidental ‘curriculum’) in direct proportion to acculturation or exposure to the Anglo, English incidental ‘classroom’” (p. 98). Their use of the term “acculturation” is a bit unfortunate, only in that it tends to refer primarily to an assimilative process reflecting the degree to which one identifies with and considers themselves a member of a particular culture. Use of the term acculturative knowledge acquisition, however, more clearly reflects Valdés’ and Figueroa’s likely intended meaning, which is related to exposure to the “incidental curriculum.” Under this definition, two individuals from the same culture cannot be assumed to be comparable as they may not have had the same experiences for the same amount of time or the same opportunity for acquisition of knowledge, language, and artifacts of that culture. Valdés and Figueroa (1994) were among the first researchers to recognize that “cultural” differences were developmental, and as long as tests measured abilities in an age-based developmental sequence, being culturally different would not illuminate any bias inherent to the test. They made this same connection to language in noting that the lack of progress in the measurement of intelligence in multilinguals “stems from the fact that ‘bilingualism’ or ‘English language proficiency’ continues to be ignored as a critical independent variable” (Valdés and Figueroa 1994, p. 99). Again, perhaps because casual definitions of bilingualism tend to refer to individuals with high levels of proficiency in two or more languages, there has been a failure by researchers to appreciate the variable as reflecting a continuum of development and learning affecting all languages to which an individual has been exposed. And because tests are arranged in a sequence reflecting age-based development, being linguistically different would also not demonstrate any issues of bias inherent to testing, with the exception of construct validity (Ortiz 2019). Valdés and Figueroa (1994) also express some dismay that “researchers have seldom noted the perplexing aspects of circumstantial bilinguals’ test data” (p. 99) and that “not even the unusual subtest profiles of bilinguals have succeeded in alerting researchers to the possible importance of the bilingual factor” (p. 100). 

**Figure 3:**
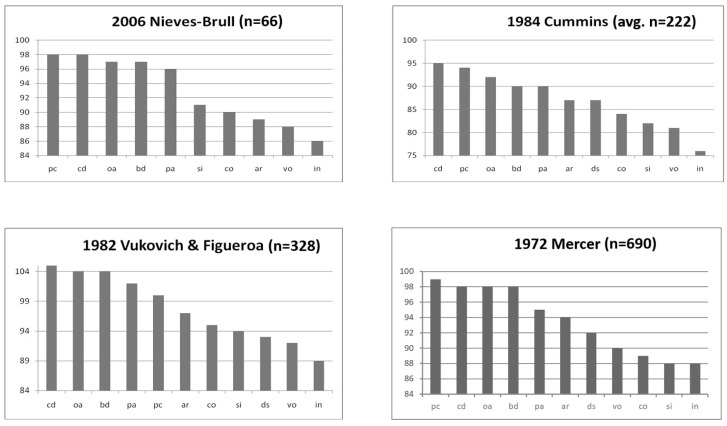
Mean WISC Subtest^3^ Performance of Multilingual Samples as Reported in Cummins (1984), Mercer (1972), Nieves-Brull (2006), and Vukovich and Figueroa (1982).

What Valdés and Figueroa (1994) alluded to in terms of “unusual subtest profiles” is made much clearer if one arranges the mean values in order of magnitude. Figure 3 shows this pattern based on four typical studies that include Mercer (1972), Vukovich and Figueroa (1982), Cummins (1984), and Nieves-Brull (2006).

Viewed from this perspective (i.e., subtests with low reliance on language/acculturative knowledge development on the left increasing to high reliance on the right), the manner in which multilinguals perform on intelligence tests given to them in English appears quite straightforward. Tests that do not require much, if any, age-appropriate language development or acculturative knowledge lead to a performance by multilinguals that is closer to or even at the monolingual normative mean. Conversely, on tests that rely on or directly measure age-appropriate language development, multilinguals perform lower and more distant from the monolingual normative mean. Although this pattern of performance mimics, to some extent, the notion of verbal vs. nonverbal test performance, the actuality is that performance is not binary and that the verbal/nonverbal distinction is an inaccurate and false dichotomy. That tests fall into one of two categories regarding the performance of multilinguals is an oversimplification, as the data show a linear and proportional effect based on the degree to which they differ from age-based normative expectations of language development. The resemblance of the studies in Figure 3 to Yerkes’ findings (shown previously in Figure 1) is uncanny and reinforces the developmental nature of language and acculturative knowledge acquisition, not merely between bilinguals and monolinguals but also between and among multilinguals. 

These principles formed the foundation of the C-LIM (Flanagan et al. 2013; Ortiz 2019), wherein subtests are organized within a framework based on the research-based means delineated in the literature from studies on multilinguals. In essence, the C-LIM takes the mean performance of multilinguals as cited in contemporary research and creates a de facto norm sample for clinical use. As such, the C-LIM is not a test and is not designed to measure anything other than the relative impact of cultural and linguistic factors on test performance. Nevertheless, it remains the only current method by which practitioners are able to apply evidence-based procedures for evaluating the validity of test scores generated on multilinguals tested with English language tests. Its singular purpose is to help practitioners ascertain the extent to which their obtained test scores may have been affected by language and acculturative knowledge factors. When cultural and linguistic factors are determined to be the primary influence on performance, the results would be considered to be likely invalid but representative of the average performance of other multilinguals with similar levels of development in English and acculturative knowledge. Conversely, when cultural and linguistic factors are determined to be absent or contributory only, the results may be deemed to be likely valid and might support inferences of disability if supported by additional evidence beyond test scores. Unlike other approaches where multilinguals are treated as if they form a homogenous group, the C-LIM allows practitioners to select the most appropriate “degree of difference” to account for developmental variation among multilinguals. In this way, the C-LIM provides three categorical research-based normative comparison standards that can be used to account not only for the attenuation of test performance relative to the use of monolingual test norms but also for attenuation that may result from differences between multilinguals in their English language development (Dynda 2008; Flanagan et al. 2013; Sotelo-Dynega et al. 2013). This latter concept has been either misunderstood by researchers or ignored and consequently undermines many of the conclusions rendered in some studies of the C-LIM. For example, in attempting to replicate the declining pattern of performance of multilinguals on the Woodcock-Johnson III: Tests of Cognitive Ability (Woodcock et al. 2001) as depicted in the C-LIM, Kranzler et al. (2010) used a convenience sample of Spanish-speakers and conducted analyses based on expectations of “typical” individuals who are “moderately different” from the mainstream and failed to recognize or appreciate how the characteristics of their sample would alter their expected performance. The researchers described their sample as comprising individuals with a mean age of 11, an average grade placement in sixth grade, and of whom the vast majority (74%) had been educated in their native language and country prior to coming to the U.S. A sample that includes such a well-educated, literate, and older school-aged population is not typically seen in studies on multilinguals where early elementary-grade students are often employed. For this reason, Kranzler et al. (2010) likely assumed their sample was much like any other sample of multilingual learners and did not account for the fact that their sample was literate in their heritage language and possessed what Cummins (1984) refers to as Cognitive Academic Language Proficiency that predisposed them toward rapid and equivalent second language acquisition and enhanced academic performance, in comparison to the more typical and younger multilinguals who arrive or are born in the U.S., enter elementary school, and are not yet literate in English, let alone their heritage language.

Other researchers have made similar errors in their investigations of test performance with multilinguals, particularly relative to expected or typical performance, which is both variable and dependent on their specific developmental characteristics. A common mistake in research has been the perception that bilinguals or multilinguals can be collectively described as encompassing a discrete, homogenous category. Doing so leads to a lack of recognition of the vast differences in development that may exist among a group of multilinguals as a function of factors such as the amount of formal education, language of instruction, socioeconomic status, disability status, and more (Ortiz 2019). For example, Styck and Watkins (2013, 2014) conducted investigations of WISC performance on multilinguals with respect to the organization of the tests within the C-LIM. They concluded that the use of the C-LIM in identifying an English learner (which is not the purpose of the C-LIM) was little more than a chance proposition as it could not distinguish them from monolinguals, which presupposes that all multilingual learners should perform similarly to each other. In contrast, the C-LIM is clear that the only pattern of performance that is common to multilinguals is that which can be denoted as average (or typical) as represented by a decline in scores as a function of increased language and acculturative knowledge demands from subtest to subset. Even then, what is average depends on the individual’s degree of difference relative to monolingual English speakers and relative to other multilinguals with more or less development in English. If a multilingual’s performance does not fall within the typical or expected range of performance for average ability, non-disabled individuals who possess the same developmental background, then the resulting pattern could be anything other than a clear decline and would, of course, be indistinguishable from the performance of any other group, including monolinguals. 

The more egregious error in the Styck and Watkins studies, however, is related to their failure to recognize that their sample was not, in fact, of average ability and non-disabled. They reported that their sample comprised 86 multilinguals, of which “roughly 97% of (n = 83) of participants were identified as meeting criteria for an educational disability (86% as SLD)” (p. 371). As noted, only multilinguals who are of average ability and who do not have a disability will display the “invalid” pattern of performance characterized by a systematic decline in scores as language and acculturative knowledge demands of the tasks increase. Given their sample, Styck and Watkins should have been looking to find the “valid” pattern of performance, not the “invalid” one, and this is precisely what they found. In their analyses, 77 subjects (89.5%) displayed the “valid” pattern (consistent with the presence of a disability). An additional three subjects (3.5%) displayed the “invalid” (consistent with average ability), which comprised the three subjects in the sample who did not have a disability. Thus, rather than undermining the credibility of the C-LIM, Styck and Watkins provided powerful data supporting the clinical utility and validity of the C-LIM by demonstrating consistency with 93% of the cases they examined (Ortiz 2019). 

That language affects test performance in a powerful and profound manner has been demonstrated since the very inception of tests and testing. The C-LIM is, at best, a stop-gap approach on the road to a more comprehensive examination of the impact of cultural and linguistic factors on the test performance of multilinguals. Nevertheless, it reinforces the importance of considering and quantifying developmental variation in language and acculturative knowledge acquisition and the manner in which they affect performance proportionally as a function of development. Errors like those made by Styck and Watkins (2013, 2014) tend to cause more confusion than illumination. Mistaken conclusions that language does not affect test performance or that multilinguals do not perform differently as compared to monolinguals or each other fly in the face of a substantive body of literature on the performance of multilinguals on tests of intelligence and cognitive ability that dates back to the work of Yerkes and the Committee (Ortiz 2019). Except in the case of highly educated bilingual and biliterate individuals, the average multilingual does not perform as well or in the same manner on any standardized test that employs norms based on monolinguals and which does not control for differences in language development and acculturative knowledge acquisition. Even multilinguals of the same age do not perform equally well as compared to each other unless they possess the same degree of development in the language of the test. The “operative factor” alluded to earlier by Yerkes (1921) is and remains language. Fairness and equity in the measurement of multilinguals must necessarily be rooted in true peer comparisons made between individuals with comparable levels of development in the language of the test and acculturative knowledge acquisition relative to the culture embedded in the test.

In summary, apart from the use of the C-LIM, practically every approach used in the evaluation of multilinguals with standardized, norm-referenced tests has been unsuccessful in helping practitioners establish test score validity. Separating tests into a verbal versus nonverbal dichotomy is too simplistic and masks the differential impact of language and acculturative knowledge acquisition on test performance. The use of nonverbal tests and test batteries ignores the centrality that language development plays in the growth and maturation of human cognitive abilities, intelligence, achievement, and language itself. Testing in the heritage language presumes equivalency in language among same-age individuals that does not exist and a degree of homogeneity of language and culture that is incorrect. Describing multilinguals as a rather monolithic group denies the tremendous variability that exists not only in English but in the heritage language as well. Language cannot be treated as something which can be overcome in the testing process. Rather, language must be viewed as something that is not only integral to the testing process but is perhaps the most important variable to be considered when constructing normative standards that will be used for age-based comparisons of performance. 

## 5. Language Difference as a Stratification Variable in Testing

Had the work of Yerkes (1921) and the Committee been more fully appreciated at the time, there might be much more to discuss with respect to the creation of tests that are valid for use with multilinguals within the context of this article. Instead, testing has wrestled with various misconceptions about the manner in which language affects test score validity, and precious little attention has been given to the creation of norms that provide true peer comparison. Apart from the recognition that the bilingual experience is not the same as the monolingual experience, the indelible stamp of the multilingual experience cannot ever be erased from an individual’s development. Comparisons of performance on any standardized, norm-referenced test, in particular those measuring intelligence, cognitive ability, or achievement, must be predicated on a group whose development and experiences are comparable to the individual being tested. This idea is not new and has been espoused many times previously. For example, Oakland and Matuszek (1976) noted that:

The acculturation patterns governing the development of many children from racial-ethnic minority groups or from lower socioeconomic homes also may be sufficiently different to warrant our judgment that the test is inappropriate…we must be sensitive to the fact that important difference exist with respect to child-rearing practices, expectations and aspirations, *language experiences*, an availability of and involvement in informal and formal learning experiences, and that these and other factors may result in acculturation patterns which are not directly comparable to those which are more typical in the United States. The decision as to whether a child’s acculturation patterns are similar to those generally reflected in the test’s standardization sample can be made individually and only after a thorough knowledge of the child’s background.

(p. 28; emphasis added)

Development experiences include language and acculturative knowledge acquisition, and those who are multilingual will likely have less of each as compared to others reared in the mainstream language and culture. More importantly, these differences are not directly related to variables that are typically included in norm samples as a way of trying to control for them. Salvia and Ysseldyke (1991) put it simply,

It must be pointed out that acculturation is a matter of experiential background rather than of gender, skin color, race, or ethnic background. When we say that a child’s acculturation differs from that of the group used as a norm, we are saying that the *experiential background* differs, not simply that the child is of different ethnic origin, for example, from the children on whom the test was standardized.

(p. 18; emphasis in original)

That language is the operative factor, as suggested by Yerkes (1921) long ago, was demonstrated further by Lohman et al. (2008) in their study examining the predictive power of nonverbal tests of intelligence on academic achievement. Apart from finding that all nonverbal tests they examined failed in predicting achievement, they noted the importance of disentangling wording that conflates racial with language differences. They stated,

Most studies compare the performance of students from different ethnic groups…rather than ELL and non-ELL children within those ethnic groups…. A major difficulty with all of these studies is that the category Hispanic includes students from diverse cultural backgrounds with markedly different English-language skills…. This reinforces the need to separate the influences of ethnicity and ELL status on observed score differences.

(pp. 276–78)

In 2014, Cormier, McGrew, and Ysseldyke investigated the degree to which performance of multilinguals on the WJ-III: COG (Woodcock et al. 2001) could be accounted for by linguistic and cultural factors primarily as a test of the subtest classifications of the WJ-III: COG within the C-LIM. Language ability was measured via a composite constructed with four subtests drawn from the co-normed achievement battery, Understanding Directions, Oral Comprehension, Story Recall, and Picture Vocabulary. Because the WJ-III: COG and WJ-III:ACH are conformed, Cormier and colleagues were able to determine the amount of variance accounted for by language on the 20 cognitive tests of the WJ-III: COG. Their results are presented in Table 1.

What jumps out quickly from the results is the linear, proportional impact that language has relative to the cognitive tests. While it is no surprise that Verbal Comprehension (VC) was explained best by language ability, given that it is a language test, it may seem odd that General Information (GI) was second. However, this finding, combined with Cormier and colleagues’ attempt to define “cultural loading”, supports the contention that culture is not related to race or ethnicity but rather is captured by the developmental acquisition of acculturative knowledge. Cormier et al. (2014) attempted to examine variance as a function of culture but ended up having to operationalize it on the basis of “(a) Foreign Born status, (b) Race, (c) Language at Home, and (d) First language (spoken at home)” (p. 614). As has been argued, neither birthplace nor race has any direct bearing on whether and to what extent an individual has been exposed to the U.S. cultural mainstream. Similarly, the inclusion of the two language variables is distinct from what should be “cultural” characteristics. Thus, it wasn’t surprising that the researchers were unable to find the same degree of impact on the test performance of multilinguals as they did with language—acculturative knowledge could not be operationalized in any independent manner other than through a measure of acculturative knowledge. Hence, GI served as an indication that language development and acculturative knowledge are perhaps two sides of the same coin, much in the way that reading and writing are almost indistinguishable under the umbrella of literacy. This is an especially important finding in that it demonstrates that acculturative knowledge is not necessarily independent of language and that the association between the two is so strong that, for practical purposes, they should be treated as measures of the same construct. What is most surprising in Table 1 is the third subtest, Concept Formation (CF), which is intended as a measure of Fluid Reasoning (Gf). However, the length and level of language used in the instructions for the administration of the task, as well as the requirement that the examinee deduce semantic relationships and express them via correct use of the conjunctions “and” and “or” seem to make the test heavily depending on language ability, as indicated by the respective percentage of variance accounted for across the age groups. 

At the bottom of the list in Table 1 are the Picture Recall (PR), Planning (P), and Spatial Relations (SR) subtests, which are primarily measures of Gv (Visual Processing) and represent tasks that require little, if any, language development. All other subtests fall in between these extremes, and the results support the linear, proportional impact of language on the test performance of multilinguals. These results converged with the work of Sotelo-Dynega et al. (2013), who had previously investigated the same concept and had found that language development (as measured by a standardized proficiency test) affected cognitive test performance on the WJ-III:COG in a proportional and continuous manner. More importantly, of the seven subtests used in their study, the extent and degree of the impact was subsequently replicated in the exact same order by Cormier et al. (2022), with the greatest impact demonstrated on the VC subtest (1st on the list in the Cormier study), followed by CF (3rd), Visual-Auditory Learning (VAL; 4th), Sound Blending (SB; 7th), Numbers Reversed (NR; 11th), Visual Matching (VM; 15th), and Spatial Relations (SR; 18th). The consistency from one study to the other highlights the robust nature of language development on cognitive test performance and its differential impact relative to the extent to which a task requires or measures language development. 

In 2022, Cormier and colleagues conducted another elegant study in which they again examined linguistic influences on cognitive test performance, in which they concluded, “Our results suggest that language abilities appear to have a significant influence on cognitive test performance” (p. 1). This conclusion truly understates their findings, however, in that they also stated that their results showed that “the influence of language ability, particularly receptive language ability, is more influential than age on cognitive test performance. This last point highlights the importance of considering language abilities when assessing students’ cognitive abilities“ (Cormier et al. 2022, p. 9). The argument has been made that age alone is an insufficient variable on which to estimate language development, which will contort normative samples and undermine their validity for use with multilinguals. Cormier and colleagues go a step further in asserting that language plays an even greater role in determining how a multilingual will perform on tests of intelligence and cognitive ability than what is generally ascribed to age. This finding was recently replicated in a study examining the need for true peer comparison of test performance, where Wong (2023) stated that lifetime English exposure “was also found to exert more influence on the variance of the raw scores on the Ortiz PVAT compared to age…and because the Ortiz PVAT measures receptive language, or specifically, receptive vocabulary, in English, the strong effect of Lifetime English Exposure above and beyond age, was observed” (p. 51).

## 6. The Bilingual English–Spanish Assessment (BESA)

At present, there are only two examples of standardized, norm-referenced tests that are based on the principles established by the research outlined in the preceding sections and which address differences in development among multilinguals. One of them comes from the field of speech-language pathology where issues of language and bilingualism have been given considerable attention and investigation, the Bilingual English–Spanish Assessment (BESA; Peña et al. 2018). The BESA is a comprehensive measure of language abilities designed for children in the preschool transition phase with ages ranging from 4:0 to 6:11 and who have been exposed to English, Spanish, or both. What is unique about the BESA, as compared to other language batteries that may be used with preschoolers, is that it recognizes that such children are not consistent in the amount of exposure or learning that has taken place in English or Spanish, even if they are of the same age. Accordingly, the BESA incorporates a screener to assist evaluators in determining the degree of development in each language so that they may be compared fairly to other multilinguals with the same amount and type of language experiences and development. Children who have virtually no exposure to Spanish comprise the “functional monolingual English” normative sample. Children who are “bilingual, dominant English” comprise their own group, which is formed on the basis that they have been exposed to more than one language, and although they are dominant in English, they are not monolingual English speakers. Children who are “balanced bilinguals” tend to have relatively equal experiences and development in English such that dominance may not even exist or be measurable in this group. Children who are “bilingual, dominant Spanish” comprise the next group and are those who have been exposed to some English but are still primarily Spanish speakers. And the final group are children who are ”functional monolingual Spanish” speakers who have had virtually no exposure to English. These five groups demonstrate a structure that reflects the continuum of language development that can exist between the monolingual endpoints. Previous test construction has focused mostly on the construction of the endpoints—monolingual samples, which are not appropriate for multilingual learners of any kind. But rather than simply create a single normative sample of multilinguals, Pena and colleagues understand the need to differentiate the development and experiences of those who may have had more English than Spanish, more Spanish than English, or perhaps a relatively equal amount of both. 

In this manner, the BESA (Peña et al. 2018) represents a standardized test that directly attempts to address the issue of language development and differences in proficiency and acculturative knowledge acquisition that are characteristic of multilingual learners. Rather than attempt to collate all English–Spanish speakers under one category (e.g., bilingual), the authors divide it into three to accommodate the fact that there are vast and significant differences between children of the same age that we may rightly call bilingual or multilingual. Age alone cannot account for differences in the lives and experiences of two children who have both been exposed to and have had some development in English and Spanish. One child may have had far more exposure to English than Spanish, and the other far less. Comparing them to each other solely on the basis of age without regard to their differential opportunities for learning would be as discriminatory as comparing the measurement of intelligence in a 5-year-old child to that of a 15-year-old child using the same standard. Age makes a difference, but so too does English language development, and current research suggests, rather startingly, that it is even more important.

## 7. The Ortiz Picture Vocabulary Acquisition Test (Ortiz PVAT)

The only other standardized, norm-referenced test that has created a norming structure based on both age and amount of English language development is the Ortiz Picture Vocabulary Acquisition Test (Ortiz PVAT; Ortiz 2018). In contrast to the BESA, the Ortiz PVAT measures receptive vocabulary only and only in English. As seen with the BESA, the introduction of a second language requires norming procedures that account for differences in development in both languages. Doing so, however, also limits the use of those norms to individuals who speak those two specific languages. Given the exceptionally large number of languages spoken in the U.S., the Ortiz PVAT was developed intentionally to measure just the English language receptive vocabulary acquisition in any individual learning English, whether monolingually from birth or as an additional language, irrespective of their heritage language. The focus on English permitted the norms to be expanded from ages 2:6 to 22:11 and provided utility for measuring English language acquisition as is typically the focus for multilinguals being evaluated in the U.S. Moreover, by limiting the test to receptive language, any individual still in the early phase of English acquisition (pre-production/silent period) can still be evaluated. 

The other major, and perhaps most significant, difference between the Ortiz PVAT and the BESA is the structure of the norming samples. Because the Ortiz PVAT only focuses on English language development, there was no need for or attempt to create a normative sample to control for developmental differences in the heritage language. As such, the test contains only two sets of norms—one for monolingual English speakers and one for non-monolingual English speakers, that is, everyone else. Creating a normative sample for monolingual English speakers (ES) is relatively easy as age serves as a suitable proxy for differences in language development. But for the English learner (EL) norms, sampling of individuals at every age had to include those with less than a month of experience with English, all the way to those who may have been learning English for nearly their entire lives, as well as a range of those in between these extremes. In this way, the Ortiz PVAT treats language development differences as the continuum that it represents rather than as a categorical variable as represented in the BESA and C-LIM. Such norming procedures are undoubtedly complicated and costly, but they are necessary for testing as they embody the principles delineated by research for establishing fairness and equity.

**Figure 4:**
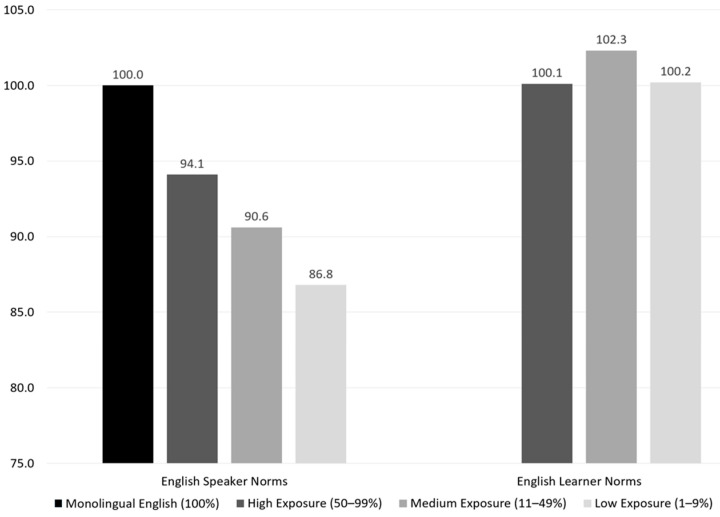
Mean Differences in Performance on the Ortiz PVAT Scored by ES norms vs. EL norms as Reported in Ortiz (2018).

That the use of exposure norms succeeds in this regard can be seen in various analyses conducted with the Ortiz PVAT. The Technical Manual (Ortiz 2018) provides an initial analysis which examines the performance of individuals who are aggregated into four categories: (1) those who are 100% monolingual English speakers and have only been exposed to English in their lives; (2) those who have had a high amount of exposure to English encompassing 50–99% of their lives; (3) those who have had a medium amount of exposure to English ranging from 11–50% of their lives; and (4) those who have had a low amount of exposure to English of less than 10% of their lives. The performance of the individuals in each group was then scored according to English speaker norms, and the three non-monolingual groups were also scored using English learner norms. Results from this analysis are presented in Figure 4.

Once again, the results on the left side of Figure 4 are uncanny in replicating Yerkes’ (1921) original analysis based on the length of residence depicted in Figure 1. When English speaker (monolingual English) norms are used, the monolingual group scores dead on average (SS = 100). However, the group with high exposure to English, in addition to another language, perform slightly lower (SS = 94), slightly more than 1/3rd SD. The medium exposure group scores lower still (SS = 90.6) than the monolingual group, which is nearly 2/3rd SD below the normative mean. And the low exposure group performs the lowest (SS = 86.8) and nearly 1SD below that of the monolingual English speakers, precisely as they did when evaluated by Yerkes (1921). Here, though, the additional analyses accomplished what Yerkes did not or could not, which is to establish that the operative factor is indeed language. Because the Ortiz PVAT developed norms to permit comparison of multilinguals of the same age and who possess the same level of English language development, it now became possible to address this question. By scoring each individual within each group against others of the same age and with the same percentage of exposure to and development in English, the resulting means for each group are found to be nearly identical. Thus, when language development is controlled, performance across all three multilingual groups is shown to both be at the normative mean of 100 as well as comparable in magnitude to each other regardless of grouping. Assuming that the sample is representative of the general population of multilinguals (apart from categorization by the amount of English development), it stands to reason that performance should be average both for the group as a whole as well as for an individual from that group when compared only to those in the same group and with the same level of development. The results in Figure 4 show demonstrate this clearly and provide powerful evidence that language, more specifically, language development differences among multilinguals, has a direct, profound, and proportional effect on intelligence and other ability test performance. 

**Figure 5:**
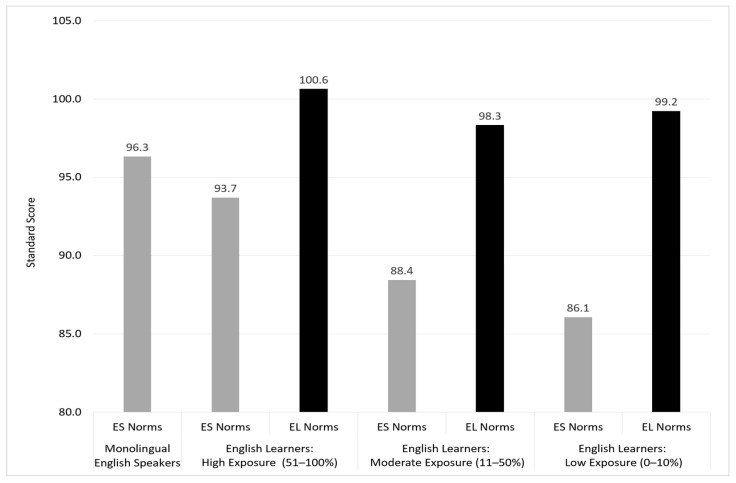
Mean Differences in Performance on the Ortiz PVAT Scored by ES norms vs. EL norms as Reported in Wong (2023).

Recently, Wong (2023) generated nearly identical results in a validation study of the Ortiz PVAT, which are presented in Figure 5. Using the same grouping variables and criteria as defined in the Ortiz PVAT Technical Manual (Ortiz 2018), Wong found the same pattern of decline in the scores as a function of English language development across the groups. Monolingual English speakers scored highest and closest to the normative mean (SS = 96.3), with statistically significant and progressive declines in the High Exposure group (SS = 93.7), to the Moderate Exposure group (SS = 88.4), and to the Low Exposure group (SS = 86.1). More importantly, Wong (2023) replicated the normatively average and comparatively equal performance of the three multilingual groups (SS = 100.6 for High Exposure, SS = 98.3 for Moderate Exposure, and SS = 99.2 for Low Exposure). 

Once more, the convergence in results from independent investigations demonstrates the robustness of the impact of language in testing. The degree and rate of decline in performance is consistent and ubiquitous and has been shown to be stable and predictable. The biggest limitation in testing has been the lack of research on the topic and the inability of researchers to generate coherent and valid studies that permit aggregation and advancement of scientific knowledge. Nevertheless, after a century of relatively minor developments in establishing fairness and equity for multilinguals, significant advances have arrived and present a renewed opportunity for the field to investigate more actively and with a greater understanding of the salient issues.

There is one final aspect of language that bears discussion at this point. In the Technical Manual of the Ortiz PVAT, an analysis was conducted on the monolingual English-speaking norm sample to evaluate differences in performance according to race. The results are presented in Table 2.

Historically, such differences have ranged from a few points to as much as 15–20 points (1.0SD to 1.5SD), with Whites scoring the highest, followed by Hispanics and Blacks (Ortiz 2019). In the monolingual English-speaking sample of the Ortiz PVAT, the developers took care to ensure that only truly monolingual English speakers were included. This meant that even individuals who may have been highly proficient in English were not included. The sample was restricted to those who had been exposed only to standard English throughout their entire lives, which usually meant fourth-generation individuals or later. When this sample was analyzed, they found no statistically significant difference between the performance of any of the four groups (White, Hispanic, Black, Other). In other words, any variance that had historically been assigned to racial or ethnic differences disappeared and strongly suggests that variance in performance has more likely been due to developmental language differences and not attributable to skin color. Note that this finding was replicated using the test’s parallel Forms A and B and with a large sample size and great power. That no difference was found, especially on a vocabulary test of the kind which had always been used to claim support for innate differences between races, once more reinforces the importance and profound impact on test performance that is right attributed to differences in language development, irrespective of race or ethnicity. 

## 8. Summary

Although the Ortiz PVAT does not measure intelligence, the structure of the test provides a blueprint for future test development of all other abilities. The principles embodied in what amounts to three-dimensional norm sample creation have roots dating back more than a century to the work described by Yerkes (1921) and the Committee in the development of examination beta. The normative structure of the Ortiz PVAT and the success it has shown empirically in establishing equivalency of performance across both racial/ethnic and linguistically diverse groups demonstrate further that Yerkes had been on the right track. It is now possible to develop a standardized, norm-referenced test that can measure any attribute, including intelligence, that exhibits fairness when used with multilinguals, irrespective of the extent to which they have developed English language proficiency, and which generates valid test scores. The key to fairness in the testing of multilinguals, as demonstrated by both current and historical research, is predicated on the creation of true peer comparison groups that directly control and account for differences in language development and acculturative knowledge acquisition. Individuals cannot be included in norm samples merely on the basis of race/ethnicity, being able to achieve a particular level of English-language proficiency, or membership in a bilingual or multilingual group. Rather, norm samples must be constructed and stratified on both age and exposure to the language of the test while encompassing the entire range of differences that can exist for each and every age for which a normative comparison is to be made. Testing can no longer retain and treat language as merely an issue of comprehension or a factor to be worked around. And norm samples cannot continue to ignore variability in language development differences among multilinguals and must endeavor instead to measure and recognize such differences as accurately as possible and not rely on general notions of bilingualism or broad categories of exposure. Cormier et al. (2022) reinforced the importance of discerning these examinee characteristics (i.e., language development) and their role in enhancing the testing process and their conclusions merit presenting them here in their entirety:

Some practitioners may have concerns regarding the additional testing time required to administer, score, and interpret performance on language ability tests. Flanagan et al. (2013) addressed this concern well, as they explained: Irrespective of whether test scores ultimately prove to have utility or not, practitioners must endeavor to ascertain the extent to which the validity of any obtained test scores may have been compromised prior to and before any interpretation is offered or any meaning assigned to them. (p. 309). Therefore, not only would this process be consistent with the aforementioned standards, but it would also lead to recommendations that are better informed and tailored to individual examinee characteristics.

(p. 10)

Both the C-LIM (Flanagan et al. 2013) and BESA (Peña et al. 2018) represent approaches that have advanced fairness in the testing of multilinguals due to their focus on assessing the language development of multilingual learners and using it as the basis for comparison of test performance. Although they rely on only three general categories of English language development, they remain consistent with research that has begun to highlight once again the importance of a multilingual’s language development on test performance. Cormier et al. (2022) reinforce this recommendation and add that “the results of our study provide an empirical basis in support of this broad recommendation” (p. 9). The Ortiz PVAT (Ortiz 2018) takes these principles and recommendations and extends them even further by permitting the comparison of individuals based on the percentage of exposure to English across their lifetime, from as little as 1% to up to 99% and every point in between. Each of these approaches is faithful to and represents extensions of fundamental principles regarding the assumption of comparability, particularly with respect to language (Salvia and Ysseldyke 1991) and are ideal exemplars of evidence-based approaches that offer promising directions for fairness in the measurement of intelligence and other abilities in multilinguals. As was learned at the very inception of testing, the need to ensure that limited English proficiency (to whatever degree) does not lead to misinterpretation of an individual’s true intellectual capabilities is fundamental to nondiscriminatory assessment and is attainable only when bilinguals are not treated like two monolinguals of the same age.

## Figures and Tables

**Table 1:** Percentage of Variance Explained by Language on WJ-III: COG Subtests by Age Group as reported by Cormier et al. (2014).

		% of Variance Explained	
	Individual Subtest	7–10	15–18
Most affected by language	Verbal Comprehension (VC)	79	81
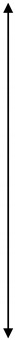	General Information (GI)	71	86
	Concept Formation (CF)	67	67
	Visual–Auditory Learning (VAL)	40	41
	Delayed Recall Visual–Auditory Learning	39	37
	Analysis Synthesis (AS)	29	47
	Sound Blending (SB)	25	35
	Auditory Working Memory (AWM)	22	32
	Retrieval Fluency (RF)	22	28
	Memory for Words (MW)	18	23
	Numbers Reversed (NR)	17	30
	Pair Cancelation (PC)	17	11
	Rapid Picture Naming (RPN)	16	16
	Incomplete Words (IW)	13	23
	Visual Matching (VM)	13	16
	Decision Speed (DS)	12	19
	Auditory Attention (AA)	10	15
	Spatial Relations (SR)	8	16
	Planning (P)	7	11
Least affected by language	Picture Recall (PR)	2	10

**Table 2:** Mean Performance Differences on the Ortiz PVAT by Racial/Ethnic Group. © 2018 Ortiz PVAT Technical Manual. Reproduced with permission from MHS, Inc. All rights reserved.

Form	Racial/Ethnic Group	*N*	*M*	*SD*	*F (df)*	*p*	Pairwise Comparisons (*p* < .01)	Partial eta^2^
**Form A**	Black	280	99.4	15.2	2.60 (3, 1523)	.051	*ns*	.005
	Hispanic	126	99.5	15.4				
	White	1018	100.5	15.3				
	Other	106	96.3	15.3				
**Form B**	Black	280	99.6	15.1	2.47 (3, 1523)	.060	*ns*	.005
	Hispanic	126	99.7	15.3				
	White	1018	100.6	15.2				
	Other	106	96.4	15.2				

## Notes

1 The terms “bilingual” and “multilingual” as used in this article are interchangeable and do not imply any particular level of proficiency. Some bilinguals/multilinguals may be highly proficient in one language but not the other, or proficient in both, or neither. The terms simply refer to someone with developmental experiences in two or more languages and is synonymous with other terms like English learner (EL), English language learner (ELL), multilingual learner (ML), all of which denote varying experiences and development with two or more languages.

2 The concept of “mental age” refers to the level at which an examinee was able to correctly complete age-based tasks comparable to the average of other individuals of a particular age group. For example, if a 10 year old examinee could do 8 year old level tasks, but no higher, then the mental age was 8. On the Stanford-Binet, average mental age for an adult male was considered to be about 13. On Goddard’s translated scale, it was about 16.

3 CD = Coding; OA = Object Assembly; BD = Block Design; PA = Picture Arrangement; PC = Picture Completion; AR = Arithmetic; CO = Comprehension; SI = Similarities; DS = Digit Span; VO = Vocabulary; IN = Information.
